# Induction of Cellular Immune Response by DNA Vaccine Coexpressing *E. acervulina* 3-1E Gene and Mature CHIl-15 Gene

**DOI:** 10.1155/2012/654279

**Published:** 2012-06-17

**Authors:** Dexing Ma, Chunli Ma, Mingyang Gao, Guangxing Li, Ze Niu, Xiaodan Huang

**Affiliations:** ^1^College of Veterinary Medicine, Northeast Agricultural University, No. 59 Mucai Street, Gongbin Road, Xiangfang District, Harbin 150030, China; ^2^College of Food Science, Northeast Agricultural University, China

## Abstract

We previously reported that the chimeric DNA vaccine pcDNA-3-1E-linker-mChIL-15, fused through linking *Eimeria acervulina* 3-1E encoding gene and mature chicken IL-15 (mChIL-15) gene with four flexible amino acid SPGS, could significantly offer protection against homologous challenge. In the present study, the induction of cellular immune response induced by the chimeric DNA vaccine pcDNA-3-1E-linker-mChIL-15 was investigated. Spleen lymphocyte subpopulations were characterized by flow cytometric analysis. The spleen lymphocyte proliferation assays were measured by 3-[4,5-dimethylthiazol-2-y1]-2,5-diphenyltetrazolium bromide (MTT) method. The mRNA profiles of ChIL-2 and ChIFN-*γ* in spleen were characterized by means of real-time PCR. Chickens immunized with pcDNA-3-1E-linker-mChIL-15 exhibited significant upregulated level of ChIL-2 and ChIFN-*γ* transcripts in spleen following two immunizations compared with chickens in other groups (*P* < 0.01). In comparison with pcDNA3.1-immunized and control groups, lymphocyte proliferation, percentage of CD8*α* ^+^ cell, and levels of ChIL-2 and ChIFN-*γ* transcripts in the group immunized with pcDNA-3-1E-linker-mChIL-15 were significantly increased on day 6 following challenge (*P* < 0.05, *P* < 0.01, and *P* < 0.01, resp.). Our data suggested that the fusion antigen 3-1E-linker-mChIL-15 could be a potential candidate for *E. acervulina* vaccine development.

## 1. Introduction

Coccidiosis, a major poultry enteric disease caused by protozoan parasites of the genus *Eimeria,* results in significant economic losses to the poultry industry [1]. Currently, although chemoprophylaxis is the predominant method used to control coccidiosis, alternate options are needed due to the emergence of drug resistance of *Eimeria* species, drug residue in animal food products, and high costs for development of new drugs [2–7]. Live vaccines and attenuated vaccines available commercially could provide partial immune protection against reinfection, but also pose the risk of unintended infection under the immunosuppressive conditions [8, 9]. It has been shown that host immunity to avian coccidiosis is largely dependent on cell-mediated immunity (CMI) [10–12], thus generating interest in developing recombinant vaccines and adjuvants that preferentially elicit cellular immune responses. Many secreted or membrane-bound proteins involved in the interaction with the host immune system have been considered as targets for immunological interventions. Some antigens located on the surface of sporozoites and schizonts have been identified as potential immune regulator and effector genes that could influence host-parasite interaction at the molecular and cellular levels [12–14]. The 3-1E antigen is on the surface of *Eimeria* sporozoites and schizonts, and the 3-1E coding sequence was reported to be conserved in several *Eimeria* spp. [15]. The DNA vaccine carrying 3-1E gene could induce immune protection against homologous challenge [16–19]. Some studies have reported that chicken cytokines, such as ChIFN-*α*, ChIFN-*γ*, ChIL-1*β*, ChIL-2, ChIL-8, ChIL-15, ChIL-18, and ChTGF-*β*4, could all enhance the host immune responses to *E*. *tenella* and* E*. *acervulina* induced by DNA vaccines [17–25]. ChIL-15 was reported to be produced by mononuclear phagocytes and other cell types in response to infection by virus or parasites, LPS, and other signals. ChIL-15 could stimulate the proliferation of NK cells [26]. Activated NK cells could produce ChIFN-*γ*, which is a major cytokine mediating resistance to *Eimeria*. Previously, we constructed a chimeric DNA vaccine, pcDNA-3-1E-linker-mChIL-15, coexpressing *E*. *acervulina* 3-1E encoding gene and mature ChIL-15 (mChIL-15) gene, and the evaluation of its protective efficacy showed that pcDNA-3-1E-linker-mChIL-15 could significantly offer protection against homologous challenge [27]. The aim of the present study was to investigate the cellular immune response induced by the chimeric DNA vaccine pcDNA-3-1E-linker-mChIL-15.

## 2. Materials and Methods

### 2.1. Birds, Parasites, and DNA Vaccine

Specific pathogen-free (SPF) White Leghorn chickens were purchased from Harbin Veterinary Research Institute (Harbin, China) and raised in wire cages in the *Eimeria*-free environment. Oocysts of *E*. *acervulina* Shanghai (SH) strain were provided by Dr. Huangbing (Shanghai Veterinary Research Institute, Shanghai, China). The sporulated oocysts were propagated in three-week-old SPF chickens and were stored in 2.5% potassium dichromate solution at 4°C [28]. The DNA vaccine pcDNA-3-1E-linker-mChIL-15 was prepared as described previously [27] and was stored in the Laboratory of Veterinary Immunopathology, Northeast Agricultural University, China.

### 2.2. Immunization and Challenge Experiment

The immunization and challenge experiments were carried on according to the previous report [27]. Briefly, seven-day-old chickens were randomly divided into three groups of 25 each. The chickens in the experimental group were, respectively, intramuscularly immunized with 100 *μ*g pcDNA-3-1E-linker-mChIL-15 (group 1) and 100 *μ*g pcDNA3.1 (group 2) at 14 days of age. Chickens in the control group (group 3) were injected with sterile TE buffer (10 mM Tris-HCl pH 7.6 and 1 mM EDTA) at the same injection site. A booster immunization was given at 21 days of age. At 28 days of age, all chickens were inoculated orally with 5 × 10^4^ sporulated oocysts of *E. acervulina* SH strain (Table 1). The challenge dose of *E*. *acervulina*-sporulated oocysts was determined according to the results of preliminary experiment in our lab.

### 2.3. Lymphocyte Proliferation Assay

Spleen samples from five chickens per group were randomly collected at 28 and 34 days of age, respectively. The samples were divided into two equal parts. One is for preparation of lymphocytes, the other is used to extract RNA for real-time PCR assay. The splenocytes were isolated on lymphocyte separation medium with a specific density of 1.007 g/mL (Tianjin Haoyang, Tianjin, China) according to the procedure as reported [29]. Briefly, the lymphocytes were collected and washed twice with culture medium (RPMI1640 containing 10% FCS, pH 7.2). Then it was finally resuspended in the culture medium at a concentration of 2 × 10^7^ cells/mL. 50 *μ*L of lymphocyte suspension was transferred into a 96-well tissue microculture plate (Costar, USA) on ice, and then 50 *μ*L concanavalin A (Con A) solution (0.02 mg/mL, Sigma) was added per well. After 24 h of culture at 39°C under 5% CO_2_, 10 *μ*L of 3-[4,5-dimethylthiazol-2-yl]-2,5-diphenyltetrazolium bromide (MTT) (5 mg/mL, Sigma) solution was added to each well and continued to incubate for 4 h. The microtiter plates were centrifuged for 5 min (1400 g, 15°C), and then 150 *μ*L of a dimethyl sulfoxide (DMSO) working solution was added to each well. The optical density (OD) of the reaction product was measured in an ELISA reader at 570 nm wavelength 15 minutes later.

### 2.4. Flow Cytometric Analysis

For evaluation of cellular immunity, single cell suspensions of spleen lymphocytes were prepared as described previously, resuspended in culture medium (RPMI1640 containing 10% FCS, pH 7.2), and adjusted to concentration of 1.0 × 10^7^ cells/mL. The cells were respectively incubated with 5 *μ*L of fluorescein-isothiocyanate- (FITC-) conjugated mouse monoclonal antibodies (mAbs) against chicken CD4^+^ (AbD Serotec) and 5 *μ*L of FITC-conjugated mouse mAbs against chicken CD8*α* ^+^ (AbD Serotec) for 30 min at room temperature. Following incubation, the cells were washed twice with PBS (pH 7.2), and the proportion of CD4^+^ and CD8*α* ^+^ cells was analyzed with 1.0 × 10^4^ viable cells using EPICS XL flow cytometer (Beckman Coulter).

### 2.5. RNA Extraction and cDNA Preparation

The spleen samples about 100 mg were homogenized with liquid nitrogen using a mortar and pestle. Total RNA was extracted from the tissue samples with 1.0 mL TRIzol reagent (Invitrogen) according to the manufacturer protocol. Prior to reverse transcription RNA was treated with RNase-free DNase (Invitrogen). Quantification of RNA was based on spectrophotometric analysis at 260/280 nm. Then, total RNA was reverse-transcribed using SYBR Premix Ex *Taq* kit (DRR037S, TaKaRa, Japan) according to the manufacture manual. The cDNA product was stored at −20°C.

### 2.6. Real-Time PCR Assay

For quantification, three kinds of standard plasmids with the cDNA fragment of ChIL-2, ChIFN-*γ* and GAPDH harbored in pMD18-T (TaKaRa, Japan) were, respectively, constructed. The standard curves of the three standard plasmids were prepared in order to relatively quantify the threshold cycles (*C*_*t*_) values of the samples. Real-time PCR was done as described by Hong et al. [14]. Triplicate quantitative assays per group were performed with an ABI prism 7000 sequence detection system. Primers and PCR conditions are described in Table 2. The negative control containing reagents only and the tenfold serial dilutions of standard plasmid with target gene fragment were included in all assays. The standard curve was generated using log_10_ diluted standard plasmid by ABI prism 7000 Sequence Detection System (SDS) v1.2.3 software. The mRNA expression level of ChIL-2, ChIFN-*γ*, and GAPDH in spleen of individual chickens was calculated using specific standard curve and was then divided by that of GAPDH of the same chicken to normalize the relative expression of ChIL-2 and ChIFN-*γ* mRNA.

### 2.7. Statistical Analyses

Data were all expressed as means ± SD and were all evaluated by variance (ANOVA). Duncan's multiple range tests were performed using Statistical Analysis System (SAS Institute, Cary, NC, USA) to analyze difference between means. Results were considered significant at *P* < 0.05 highly significant at *P* < 0.01.

## 3. Results

### 3.1. Effect of Immunization with pcDNA-3-1E-Linker-mChIL-15 on Spleen Lymphocyte Proliferation

**Figure 1:**
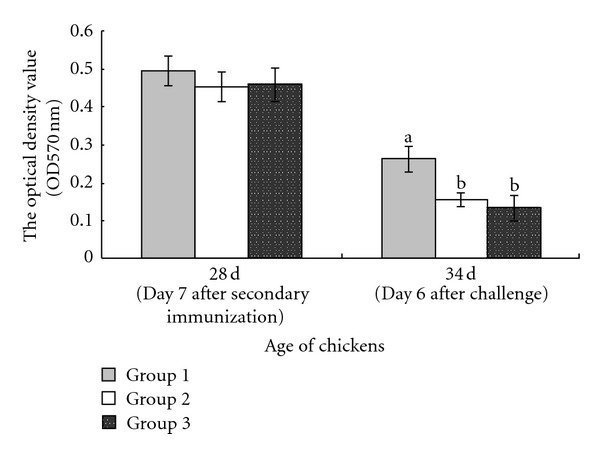
Effect of immunization with pcDNA-3-1E-mChIL-15 on spleen lymphocyte proliferation. Seven-day-old chickens were divided into three groups. Chickens in each group were, respectively, intramuscularly immunized with 100 *μ*g pcDNA-3-1E-linker-mChIL-15 (group 1), 100 *μ*g pcDNA3.1 (group 2), and sterile TE buffer (pH 7.6) (group 3) at 14 days of age. A booster immunization was given at 21 days of age. All chickens were inoculated orally with 5 × 10^4^ sporulated oocysts of *E. acervulina* at 28 days of age (day 7 after secondary immunization). The proliferative response of spleen lymphocytes to stimulation with Con A was measured by MTT methods at day 28 and 34 days of age (day 6 after challenge). Data were expressed as means ± standard errors (*n* = 5 per group). Bars not sharing the same small letter are significantly different according to Duncan's multiple range test (*P* < 0.05) in each time point.

The spleen lymphocyte proliferation response to stimulation with Con A measured by MTT method is described in Figure 1. The proliferation response in the group immunized with pcDNA-3-1E-linker-mChIL-15 (group 1) was higher than that in the other two groups at 34 days of age (on day 6 after challenge) (*P* < 0.05).

### 3.2. Effect of Immunization with pcDNA-3-1E-Linker-mChIL-15 on Spleen Lymphocyte Subpopulations

**Figure 2:**
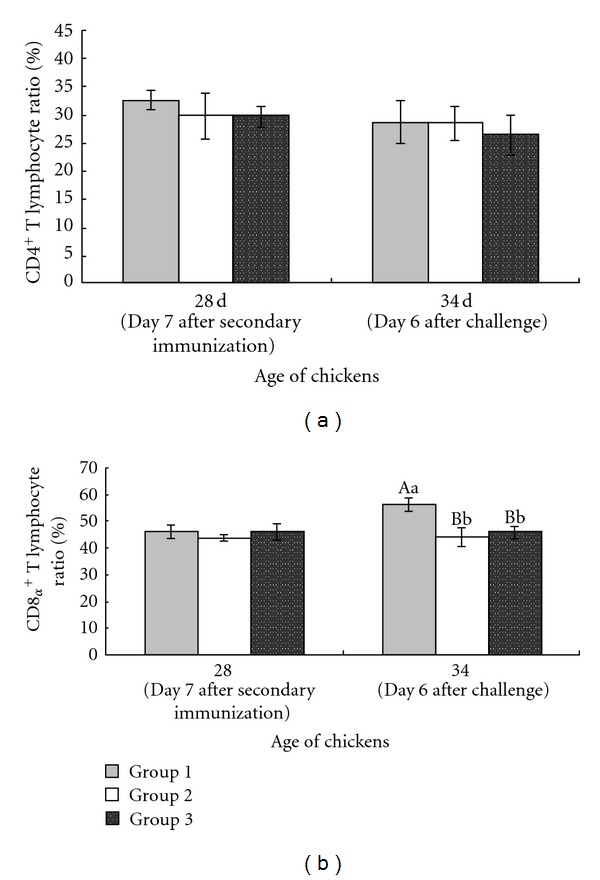
Flow cytometric analysis of CD4^+^ and CD8*α* ^+^ cells isolated from splenocytes of chickens. Chickens in each group were, respectively, intramuscularly immunized with 100 *μ*g pcDNA-3-1E-linker-mChIL-15 (group 1), 100 *μ*g pcDNA3.1 (group 2), and sterile TE buffer (pH 7.6) (group 3) at 14 and 21 days of age. All chickens were inoculated orally with 5 × 10^4^ sporulated oocysts of *E. acervulina* at 28 days of age (day 7 after secondary immunization). The ratio of CD4^+^ and CD8*α* ^+^ cells in spleen of randomly selected five chickens was assayed at 28 and 34 days of age (day 6 after challenge). Data represent mean ± standard errors (*n* = 5 per group). Highly significant difference (*P* < 0.01) between bars not sharing the same capital letters in each time point. Significant difference (*P* < 0.05) between bars not sharing the same small letters in each time point.

Chicken lymphocytes have an important role in the host immune response to *Eimeria* infection. Therefore, we measured the changes of spleen lymphocyte subpopulations in chickens on day 7 after secondary immunization (28 days of age) and on day 6 after challenge (34 days of age). Chickens in the group immunized with pcDNA-3-1E-linker-mChIL-15 exhibited significant increased percentage of CD8*α* ^+^ cells compared with the other two groups on day 6 after challenge (*P* < 0.01) (Figure 2).

### 3.3. Effect of Immunization with pcDNA-3-1E-Linker-mChIL-15 on Splenic Cytokine mRNA Level

**Figure 3:**
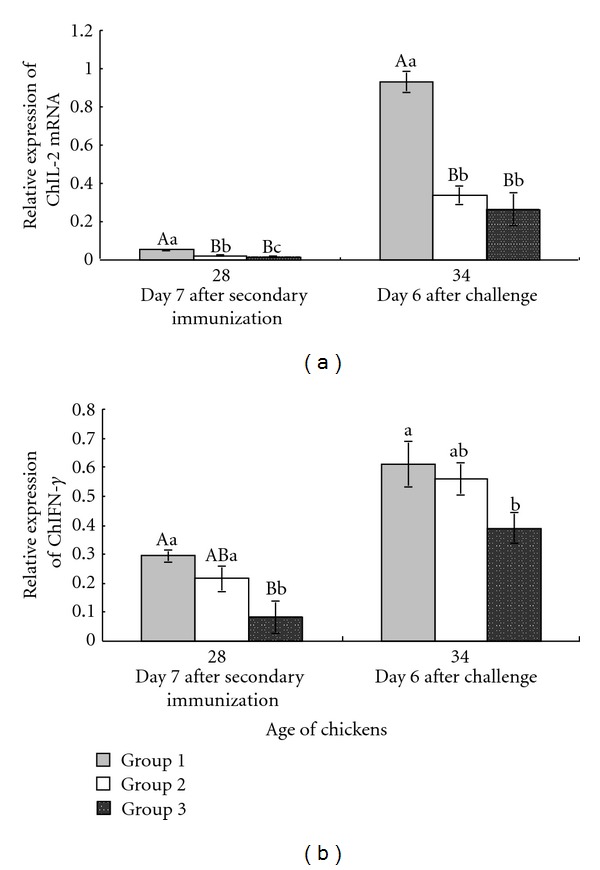
Effect of immunization with pcDNA-3-1E-mChIL-15 on ChIFN-*γ* and ChIL-2 mRNA levels. Seven-day-old chickens were divided into three groups. Chickens in each group were, respectively, intramuscularly immunized with 100 *μ*g pcDNA-3-1E-linker-mChIL-15 (group 1), 100 *μ*g pcDNA3.1 (group 2), and sterile TE buffer (pH 7.6) (group 3) at 14 and 21 days of age. All chickens were inoculated orally with 5 × 10^4^ sporulated oocysts of *E. acervulina* at 28 days of age (day 7 after secondary immunization). The level of splenic mRNAs encoding ChIFN-*γ* and ChIL-2 was measured by real-time PCR at 28 and 34 days of age (day 6 after challenge). The cytokine mRNA levels of individual chicken in each group were divided by mRNA levels of GAPDH of the same chicken to normalize the relative mRNA levels of ChIL-2 and ChIFN-*γ*. Data were expressed as mean ± standard errors (*n* = 5 per group). Highly significant difference (*P* < 0.01) between bars not sharing the same capital letters in each time point. Significant difference (*P* < 0.05) between bars not sharing the same small letters in each time point.

The level of mRNA for ChIL-2 and ChIFN-*γ* in spleen of chickens in each group was illustrated in Figure 3. Following two immunizations (at 28 days of age), chickens immunized with pcDNA-3-1E-linker-mChIL-15 (group 1) showed significant increased level of transcripts for ChIL-2 and ChIFN-*γ* compared with chickens in pcDNA3.1-immunized and nonimmunized groups (*P* < 0.01). On day 6 after challenge (at 34 days of age), the level of transcripts for cytokines in group 1 was significantly higher (*P* < 0.01) for ChIL-2 and higher (*P* < 0.05) for ChIFN-*γ* compared with that in group 2 and group 3.

## 4. Discussion

It has been well documented that DNA immunization could elicit protective immunity against pathogenic parasites, including *Eimeria* sp., *Leishmania* sp., and *Toxoplasma gondii* [27, 30–32]. Several important antigens existing in sporozoites and schizonts including 3-1E and EtMIC2 [17, 33], SO7 [30], TA4 [34], 5401 [35], EASZ240 and EASZ250 [36], and rhomboid-like protein [37] have already been evaluated to be immunogenic. 3-1E antigen was presented on the outer surface of both sporozoites and merozoites. The 3-1E gene as DNA vaccine candidate and a series of chicken cytokine as adjuvant of DNA vaccine have been reported [18, 19, 24]. We also successfully constructed a novel chimeric DNA vaccine, pcDNA-3-1E-linker-mChIL-15, coexpressing *E. acervulina* 3-1E gene and mature ChIL-15 (mChIL-15) in our previous studies for the first time, and the animal experiments showed that the chimeric DNA vaccine could offer protection against homologous challenge, including significantly increasing oocyst decrease ratio, reducing the average lesion score in the duodenum, improving body weight gain, and increasing anticoccidial index (ACI) [27]. However, the cellular immune response elicited by chimeric DNA vaccine coexpressing 3-1E gene and mChIL-15 has not been reported until now. So the purpose of the present study was to elucidate the cellular immune response induced by pcDNA-3-1E-linker-mChIL-15.

On day 7 following two immunizations, the significant increased level of mRNA for ChIL-2 and ChIFN-*γ* in spleen was observed in group 1 compared with group 2 and 3 (*P* < 0.01). The above results may be explained by the following aspects. In our previous report, the expression of 3-1E-linker-mChIL-15 fusion proteins was easily observed in vitro in the cytoplasm of transfected 293T cells by indirect fluorescent antibody test [27]. This suggested that the antigen 3-1E-linker-mChIL-15 could be naturally processed and targeted to the major histocompatibility complex class I (MHC I) binding pathway [38].

Analysis of lymphocyte subpopulation in spleen including CD4^+^ and CD8*α* ^+^ provides important support for measuring the induction of cellular immune response after immunization with pcDNA-3-1E-linker-mChIL-15. On day 6 after challenge, the chickens immunized with pcDNA-3-1E-linker-mChIL-15 displayed significant increased spleen lymphocyte proliferation and percentage of CD8*α* ^+^ cell (*P* < 0.05, *P* < 0.01, resp.). It is generally accepted that the cell-mediated immunity (CMI) in particular mediated by CD8*α* ^+^ cytotoxic T lymphocytes plays a critical role in mediating protective immunity during avian coccidiosis [39]. The percentage of CD8*α* ^+^ cells in spleen may partially reflect the enhanced cellular immune status against *Eimeria* infection.

The host cell immune response to *Eimeria* infection could be initiated, immunoregulated, and amplified through cytokines net [14]. On day 6 after challenge, the significantly increased ChIL-2 and ChIFN-*γ* transcripts (*P* < 0.01, and *P* < 0.05, resp.) in group 1 were observed. ChIFN-*γ* is an important cytokine mediating protective immune response to avian coccidiosis and has been shown to inhibit *E. tenella* development in vitro [23, 40]. ChIL-2 could selectively enhance the differentiation and development of Th1 type cells that could conversely induce the secretion of ChIFN-*γ* and ChIL-2. It has been reported that a significant increase of ChIL-2 mRNA transcripts was observed in the spleen and intestine after primary and secondary infections with *E*. *acervulina* [41]. On day 6 after challenge, the *E*. *acervulina* has completed its intracellular life cycle and is excreted. Therefore, the above results in this study suggested that the cellular immune response against *E*. *acervulina* infection was effectively induced and the invasion of sporozoites or merozoites into the gut epithelial cells was blocked by immunization of DNA vaccine pcDNA-3-1E-linker-mChIL-15. All the above results and analysis are in accordance with the previous observation of protection efficacy against homologous challenge, such as increasing oocyst decrease ratio and reducing the average lesion score in the duodenum [27]. The fusion protein 3-1E-linker-mChIL-15 was fused through linking 3-1E protein and mChIL-15 protein with four flexible amino acid SPGS, which could make the two polypeptide fragments and side chains of amino acids fold correctly to produce a spatial structure with biological activity and function. To further explore the detailed mechanism of cellular immune response induced by fusion protein 3-1E-linker-mChIL-15, we prepare to design more experimental groups in our subsequent research, containing pcDNA3-1E group, pcDNAChIL-15 group, and pcDNA3-1E coinjected with pcDNAChIL-15 group.

Recently, several studies have investigated the important role of TNF superfamily (such as TNFSF-15) and IL-17 family (such as IL-17F) in the regulation of immune response during infection of *Eimeria* [42–44]. In addition, it was reported that the antibody-mediated immunity also played roles against the *Eimeria* infection [45, 46]. So, one of the current works in our laboratory is directed at investigating the more detailed immune mechanisms of the novel chimeric DNA vaccine pcDNA-3-1E-linker-mChIL-15 including humoral immunity and innate immunity such as the Toll-like receptor (TLR) signal pathway.

In conclusion, our data indicated that the cellular immune response for administration of 3-1E-linker-mChIL-15 involved significant increased lymphocytes proliferation, percentage of CD8*α* ^+^ cells, and mRNA level of ChIL-2 and ChIFN-*γ* in spleen on day 6 after challenge.

## Figures and Tables

**Table 1:** Experimental groups used in immunization and challenge experiments.

Group	Number	Immunization		Challenge at 28 days of age
		Primary immunization at 14 days of age	Secondary immunization at 21 days of age	Number of *E*. *acervulina-sporulated oocysts *
1	25	pcDNA-3-1E-mChIL-15 (100 *μ*g)	pcDNA3-1E-mChIL-15 (100 *μ*g)	5 × 10^4^
2	25	pcDNA3.1 (100 *μ*g)	pcDNA3.1 (100 *μ*g)	5 × 10^4^
3	25	TE buffer (pH 7.6)	TE buffer (pH 7.6)	5 × 10^4^

**Table 2:** Primer sequences and PCR conditions. The oligonucleotides were used to analyze the relative expression level of ChIL-2 and ChIFN-*γ* mRNA.

mRNA target	GeneBank accession no.	Primer sequence	PCR product Size (bp)	Annealing temperature. (°C)
ChIL-2	NM204153	F 5′-GTGGCTAACTAATCTGCTGTCC-3′	105	55.5
		R 5′-GTAGGGCTTACAGAAAGGATCAA-3′		

ChIFN-*γ*	NM205149	F 5′-CAAAGCCGCACATCAAACAC-3′	80	56.0
		R 5′-tTTCACCTTCTTCACGCCATC-3′		

ChGAPDH	NM204305	F 5′-AGAACATCATCCCAGCGTCC-3′	133	57.5
		R 5′-CGGCAGGTCAGGTCAACA-3′		
